# Screening circulating proteins to identify biomarkers of fetal macrosomia

**DOI:** 10.1186/s13104-019-4625-1

**Published:** 2019-09-18

**Authors:** Tess Cruickshank, Tu’uhevaha J. Kaitu’u-Lino, Ping Cannon, Alesia Harper, Tuong-Vi Nguyen, Kirsten M. Dane, Anna L. Middleton, Valerie P. Kyritsis, Roxanne Hastie, Stephen Tong, Susan P. Walker, Teresa M. MacDonald

**Affiliations:** 1 Translational Obstetrics Group, The Department of Obstetrics and Gynaecology, Mercy Hospital for Women, University of Melbourne, 163 Studley Road, Heidelberg, Melbourne, VIC 3084 Australia; 2 Mercy Perinatal, Mercy Hospital for Women, Heidelberg, Melbourne, VIC Australia

**Keywords:** Biomarker, Macrosomia, Plasma, Pregnancy

## Abstract

**Objective:**

Fetal macrosomia is a major risk factor for shoulder dystocia, which can lead to birth asphyxia, maternal and neonatal traumatic injuries, and perinatal death. If macrosomia is diagnosed in the antenatal period, labour can be induced to decrease shoulder dystocia. But current clinical methods to diagnose fetal macrosomia antenatally perform with poor accuracy. Therefore, improved methods to accurately diagnose fetal macrosomia are required. Blood biomarkers that predict fetal macrosomia could be one such novel diagnostic strategy. We undertook a nested case–control study from a prospective collection of 1000 blood samples collected at 36 weeks’ gestation. We analysed plasma samples from 52 women who subsequently delivered a macrosomic (> 95th centile for gestational age) infant and 106 controls. Circulating concentrations of the proteins COBLL1, CSH1, HSD3B1, EGFL6, XAGE3, S100P, PAPPA-1, ERBB2 were assessed for their ability to predict macrosomic infants.

**Results:**

We did not identify any significant changes in the plasma concentrations of COBLL1, CSH1, HSD3B1, EGFL6, XAGE3, S100P, PAPPA-1, ERBB2 from women who subsequently delivered macrosomic neonates relative to control samples. Although we have not identified any potential biomarkers of fetal macrosomia, we have ruled out these particular eight protein candidates.

## Introduction

Fetal macrosomia is a risk factor for birth complications including operative vaginal delivery, emergency caesarean section, and shoulder dystocia [1, 2]. The incidence of shoulder dystocia increases with increasing birthweight [1, 3–5], such that around half of all shoulder dystocia cases occur in infants considered to be large-for-gestational-age [3]. For infants, macrosomia and shoulder dystocia can predispose to brachial plexus injury, facial nerve injuries, fractures to the humerus and birth asphyxia [6]. Meanwhile, mothers also carry increased risk of postpartum haemorrhage and significant perineal trauma [1, 5, 7]. Women who are overweight or obese have a higher risk of having a macrosomic neonate [8]. Given that obesity is increasing, this is a major public health issue [9].

Recently, a randomised controlled trial was performed comparing induction of labour to expectant management for cases where a macrosomic fetus was suspected clinically, and subsequently found to have an ultrasound estimated fetal weight (EFW) of > 95th centile for gestational age [10]. This study found that induction of labour, compared to expectant management, significantly reduced the risk of shoulder dystocia or associated morbidity, with a relative risk of 0.32. There was also an increased rate of spontaneous vaginal deliveries with induction of labour [10]. Unfortunately, antenatal diagnosis of fetal macrosomia with clinical examination and ultrasound is low in accuracy [11]. Detecting macrosomia through ultrasound alone has a 10–15% error margin [12, 13] and clinical methods such as symphysis-fundal height measurement also demonstrate low predictive values [14]. Both methods suffer from low sensitivity due to variables including maternal adipose tissue, and inter-operator variability [6]. Moreover, failure to diagnose fetal macrosomia can lead to increased maternal and neonatal complications [15].

A more precise diagnostic procedure is needed if we are to effectively diagnose macrosomic fetuses in order to reduce their risk of shoulder dystocia and associated maternal and neonatal morbidity. Therefore, this study investigates the potential of blood-based biomarkers for macrosomia, as part of the Fetal Longitudinal Assessment of Growth (FLAG) study. The aim of this study was to assess eight proteins within the maternal plasma at 36 weeks’ gestation for their capacity to predict subsequent fetal macrosomia, with the hope of identifying new biomarkers of this condition.

## Main text

### Materials and methods

#### Study overview

This is a sub-study of the Fetal Longitudinal Assessment of Growth (FLAG) study, which was undertaken at a tertiary maternity hospital in Melbourne, Australia—the Mercy Hospital for Women. The FLAG study prospectively collected blood samples from 2015 pregnant women at 28 and 36 weeks’ gestation. We screened women for eligibility and invited them to participate at the time of their attendance for the routine pregnancy oral glucose tolerance test, performed to screen for gestational diabetes mellitus at around 28 weeks’ gestation. Women were eligible to participate if they spoke English, were aged over 18 years, were carrying a singleton pregnancy, and had had a normal mid-trimester fetal morphology ultrasound assessment. Participants donated the study blood samples (whole blood collected in a 10 ml ethylenediaminetetraacetic acid tube) at between 27^+0^ and 29^+0^ weeks’ and/or between 35^+0^ and 37^+0^ weeks’ gestation inclusive. Plasma was stored at − 80 °C until sample analysis was performed.

This study was approved by the Mercy Health Research Ethics Committee (Ethics Approval Number R14/12) and written informed consent was obtained from all participants.

#### Outcomes and definitions of cases

A single clinician, blinded to all protein levels, phenotyped the participant characteristics and the outcomes of the pregnancy. This was achieved by review of the participant’s medical records, investigation results and birthing outcome summary.

We used the GROW software^23^ (http://www.gestation.net), which generates a ‘term optimal weight’ based on an optimised fetal weight standard, to assign customised infant birthweight centiles. This was used to adjust for maternal height and parity; infant sex; and exact gestational age—deemed to be non-pathological contributors to fetal growth potential. We did not adjust for maternal weight or ethnicity. A local dataset was used to generate coefficients for the Australian dataset of GROW. For each of the adjusted variables, the model has a constant to which weight is added or subtracted. Macrosomia was defined as customised infant birthweight > 95th centile (because of the data demonstrating reduced shoulder dystocia with induction of labour at this threshold [10]).

#### Power calculation and selection of samples for analysis

Given that our definition of macrosomia was > 95th centile, we estimated a prevalence of 5%. We calculated that 48 cases would be needed, with two controls per case, to achieve 80% power to detect a fivefold increased odds of macrosomia with a positive test. This therefore required a total cohort of 960 from which to select 48 cases and 96 controls. Given that we had over 2000 FLAG participants in total, we limited our analysis to the first 1000 samples.

A nested case–control set of samples from the first 1000 FLAG participants who donated blood at 36 weeks’ gestation was used. Among the first 1000 FLAG participants, there were 52 (5.2%) cases of macrosomia with infant birthweight > 95th centile. These were all analysed and compared to 106 control samples. Controls were randomly selected from the first 1000 FLAG participants in order to represent the characteristics of the entire cohort.

#### ELISA analysis of circulating placental proteins in maternal plasma

##### Sandwich ELISAs

ErbB2 (Receptor tyrosine kinase 2) and PAPPA-1 (Pappalysin-1) were measured using the Human ErbB2/Her2 DuoSet ELISA kit and the Human Pappalysin-1 DuoSet ELISA kit (Minneapolis, USA) according to manufacturer’s instructions. XAGE3 (X Antigen Family Member 3), S100P (Calcium Binding Protein P), CSH1 (Chorionic Somatomammotropin Hormone 1) and EGFL6 (Epidermal Growth Factor-Like Protein 6) were measured using the Human G Antigen Family D Member 4 ELISA kit, Human S100 Calcium Binding Protein P ELISA kit, Human CSH1/Placental ELISA kit and the Human Epidermal Growth Factor-Like Protein 6 (by MyBioSource, San Diego, USA) according to manufacturer’s instructions.

##### Competitive ELISA

COBLL1 and HSD3B1 were measured using the Human Cordon Bleu Protein Like 1 ELISA kit, and the Human HSD3B1 (3 beta-hydroxysteroid dehydrogenase/Delta 5 → 4-isomerase type 1) ELISA kit (MyBioSource, San Diego, USA) according to manufacturer’s instructions (see Table 1 for details of source, dilutions and detection ranges).

**Table 1:** ELISAs inter-assay CV, dilution and detection rates for COBLL1 (Cordon-Bleu Protein-Like 1), CSH1 (Chorionic Somatomammotropin Hormone 1), HSD3B1 (Hydroxy-Delta-5-Steroid Dehydrogenase), EGFL6 (Epidermal Growth Factor-Like Protein 6), XAGE3 (X Antigen Family Member 3), S100P (Calcium Binding Protein P), PAPPA-1 (Pappalysin-1), ERBB2 (Receptor tyrosine kinase 2)

Protein	Company	Inter-assay CV or intra?	Dilution	Detection range (pg/ml)
COBLL1	myBiosource	Inter-assay < 10% Intra-assay < 10%	Neat	5000–100,000
sCSH1	myBiosource	Inter-assay CV < 10% Intra-assay CV < 10%	1:20	2500–160,000
HSD3B1	myBiosource	Inter-assay CV < 10% Intra-assay CV < 8%	1:200	313–10,000
EGFL6	myBiosource	Inter-assay CV < 10% Intra-assay CV < 8%	Neat	78–5000
XAGE3	myBiosource	Inter-assay CV < 15% Intra-assay CV < 15%	Neat	250–8000
S100P	myBiosource	Inter-assay CV < 10% Intra-assay CV < 8%	Neat	78–5000
PAPPA-1	R&D	N/A	1:40	781–50,000
ERBB2	R&D	N/A	1:20	54.7–3500

#### Statistical analysis

Data was tested for normal distribution and statistically analysed as appropriate. If the continuous data was normally distributed a parametric unpaired t-test was used. If the data was not normally distributed a Mann–Whitney U test was used. Categorical data was analysed with Fisher’s exact test. For BMI, which was significantly different between case and control groups, regression analyses including BMI as a covariate were undertaken. Statistical analyses were performed using GraphPad Prism version 6 (GraphPad Software Inc., San Diego, CA).

### Results

The maternal characteristics and pregnancy outcomes from the macrosomia cases and controls are summarised in Table 2. Women birthing infants with macrosomia (birthweight > 95th centile) were of higher BMI (median 28.3 compared to 23.5), and were more often delivered by caesarean section. They also delivered their infants an average of 2 days earlier. Unsurprisingly, their babies were much bigger in absolute birthweight, and birthweight centile, given that that was the basis of their categorisation as cases.

**Table 2:** Maternal characteristics and pregnancy outcomes for macrosomia cases compared to controls

	Macrosomia N = 52	Controls N = 106	*p*
Age	33.8 (6.1)	32.6 (4.0)	0.15
Booking BMI	28.3 [24.1–33.7]	23.5 [21.4–26.3]	< 0.0001
Nulliparous	15 (28.8%)	46 (43.4%)	0.08
Smoking status			
Current smoking	1 (1.9%)	1 (0.9%)	0.77
Ex-smoker	21 (23.1%)	21 (19.8%)	
Never smoked	39 (75%)	84 (79.2%)	
GDM	4 (7.7%)	9 (8.5%)	1.00
Onset of labour			
Spontaneous	20 (38.5%)	53 (50.0%)	0.10
Induced	13 (25.0%)	31 (29.2%)	
No labour	19 (36.5%)	22 (20.8%)	
Mode of delivery			
Physiological vaginal	21 (40.4%)	53 (50.0%)	0.002
Instrumental delivery	2 (3.8%)	21 (19.8%)	
Caesarean section	29 (55.8%)	32 (30.2%)	
Gestation at delivery (weeks^+days^)	39^+2^ (1^+0^)	39^+4^ (1^+1^)	0.04
Birthweight (g)	4352 (348.1)	3487 (406.3)	< 0.0001
Birthweight centile	97.9 [96.5–99.4]	47.1 [26.5–67.6]	< 0.0001

Data presented as mean (standard deviation) if normally distributed data, as median [interquartile range] if not normally distributed data, and as number (%) if categorical. Some percentages do not sum to 100% due to rounding to one decimal place

*BMI* Body Mass Index, *GA* gestational age; *GDM* gestational diabetes mellitus

#### Selection of proteins for measurement

The eight proteins analysed were chosen after referencing two online data repositories, as being: (i) highly expressed in the placenta relative to all other human tissues (BioGPS); and (ii) expressed on the placental surface that abuts the maternal circulation (syncytiotrophoblast; Human Protein Atlas). Further to this, XAGE3, S100P, CSH1, ErbB2, PAPPA1, EGLF6, COBLL1 were all identified as having potential roles in growth and development, whilst XAGE3, ErbB2, PAPPA1, EGLF6 have reported links to tumour growth [16–22]. HSD3B1 catalyzes the conversion of delta-5-3-beta-hydroxysteroid precursors into delta-4-ketosteroids, which is the precursor to all classes of steroid hormones [16, 23].

**Fig. 1:**
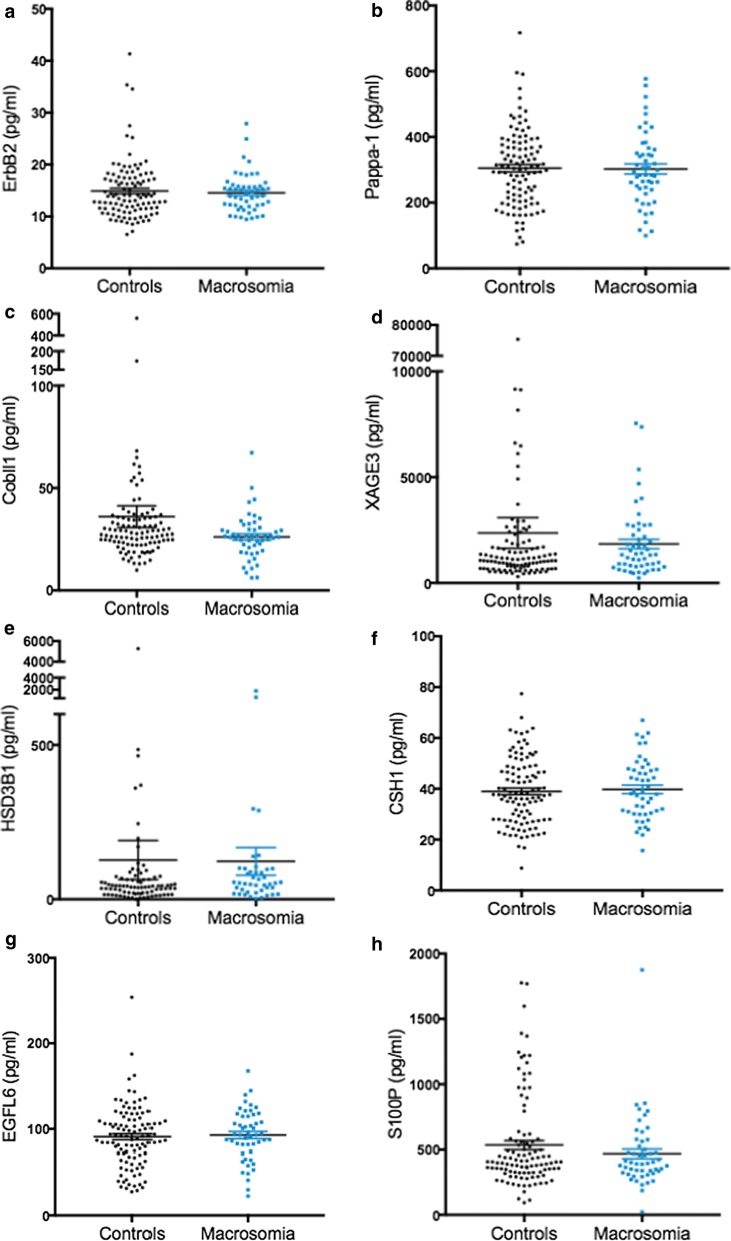
No significant difference seen between circulating proteins in controls compared to participants with macrosomic neonates. ErbB2 (**a**), PAPPA-1 (**b**), COBLL1 (**c**), XAGE3 (**d**), HSD3B1 (**e**), CSH1 (**f**), EGLF6 (**g**), S100P (**h**) compared across control plasma and the plasma of patients who were carrying macrosomic neonates. Data expressed as mean ± SEM (pg/ml), with symbols representing individual patients

We successfully measured XAGE3, S100P, CSH1, ErbB2, PAPPA1, EGLF6, COBLL1 and HSD3B1 in the maternal circulation at 36 weeks and all samples were detected within the region of the standard curve. Disappointingly however, we found no significant changes in any of the proteins in women carrying a macrosomic fetus relative to controls (Fig. 1). When we considered BMI as a covariate (given it was significantly different between groups), we still found no significant difference in protein expression between macrosomia patients and controls (data not shown).

### Discussion

In this study, we sought to identify new markers of fetal macrosomia at 36 weeks’ gestation using a case–control cohort from a large prospective collection of plasma samples. This study featured well-characterised participants and pregnancies, and a large number of macrosomia cases. Although we did not find any significant changes, our study has been useful in ruling out eight candidate proteins as blood biomarkers for fetal macrosomia.

The proteins we analysed were selected because they are highly expressed in the placenta and all localise to the syncytiotrophoblast. Thus, we reasoned they would likely be released from the placenta into the maternal circulation. Many of these proteins have been barely studied in placenta (such as XAGE-3, and COBLL1), while others have well reported roles in placental function. For example, CSH1 (or placental lactogen) has an important role in growth control [16], whilst ErbB2 encodes an epidermal growth factor receptor that abundantly localises to the placental surface and reportedly plays important roles in placental function [24]. Similarly, PAPPA1 is involved with insulin-like growth factor binding protein cleavage resulting in the insulin-like growth factor pathway activation [25]. It also has a role in bone formation and female fertility [16]. PAPPA-1 has a biased expression in the placenta [16].

## Limitations

A major limitation of this study is that we have not screened all candidate proteins (those highly expressed by the placenta, localised to the syncytiotrophoblast layer and involved in tissue growth) for their ability to predict macrosomia. Secondly, this study is not powered for the main clinical outcome we would aim to reduce—shoulder dystocia itself, but our aim is to identify fetal macrosomia—the greatest risk factor for this significant clinical outcome.

Identification of new biomarkers that could accurately predict fetal size and macrosomia could reduce both the maternal and fetal complications associated with macrosomia and shoulder dystocia. Although we were unable to detect any differences in the eight proteins we measured, other circulating proteins of placental origin may hold the key to improved detection of women and babies at risk.

## Data Availability

The datasets used and analysed during the current study are available from the corresponding author on reasonable request.

## Abbreviations

COBLL1: Cordon-Bleu Protein-Like 1
CSH1: Chorionic Somatomammotropin Hormone 1
HSD3B1: Hydroxy-Delta-5-Steroid Dehydrogenase
EGFL6: Epidermal Growth Factor-Like Protein 6
XAGE3: X Antigen Family Member 3
S100P: Calcium Binding Protein P
PAPPA-1: Pappalysin-1
ERBB2: Receptor tyrosine kinase 2
